# Supplementary material for: Light‐Induced Quantum Anomalous Hall Effect on the 2D Surfaces of 3D Topological Insulators
Source: Adv Sci (Weinh). 2021 Jul 2;8(17):2101508. doi: 10.1002/advs.202101508 (PMC8425926; doi:10.1002/advs.202101508)
Supplement: Supplementary file 1 — Supporting Information [file ADVS-8-2101508-s001.pdf]

© 2021 Wiley-VCH GmbH

Supporting Information

for *Adv. Sci.*, DOI: 10.1002/advs.202101508

Floquet quantum anomalous Hall effect on the surfaces of topological insulators

Haowei Xu, Jian Zhou, and Ju Li

Supplementary Information
of
Floquet quantum anomalous Hall effect on the surfaces of topological insulators

Haowei Xu[1], Jian Zhou[1], and Ju Li[1,2]

[1]Department of Nuclear Science and Engineering, Massachusetts Institute of Technology, Cambridge, Massachusetts 02139, USA

[2]Department of Materials Science and Engineering, Massachusetts Institute of Technology, Cambridge, Massachusetts 02139, USA

1 Methods

1.1 *Ab initio* calculations

The first-principles calculations in this work are based on density functional theory (DFT) [1, 2] implemented in Vienna ab initio simulation package (VASP) [3, 4]. The exchange-correlation interactions are treated by generalized gradient approximation (GGA) in the form of Perdew-Burke-Ernzerhof (PBE) [5]. Core electrons are treated with projected augmented wave (PAW) method [6], while the valence electrons are treated by a plane wave basis set with cutoff energy of 300 eV for Bi_2Se_3. The first Brillouin zone is sampled by a $13 \times 13 \times 5$ Γ-center k-mesh for the conventional standard unitcell of Bi_2Se_3.

1.2 Tight-binding Hamiltonian

The Bloch wavefunctions from DFT calculations described above are then used to build a tight-binding Hamiltonian with the Wannier90 package [7]. The atomic orbitals are defined with

$$|nR\rangle = \frac{1}{N}\sum_{k} e^{-\mathrm{i}k\cdot R}\sum_{m=1}^{J} U_{mn}^{k}|mk\rangle \tag{S1}$$

where $|mk\rangle$ is the Bloch wavefunctions from DFT, R are Bravais lattice vectors, J is the number of atomic orbitals in a unit cell. We use the p_x, p_y and p_z orbitals of Bi and Se to build the tight-binding Hamiltonian. U_{mn}^{k} is a unitary transformation. Note that when using Wannier90 we did not minimize the spread the wavefunctions (by setting num_iter = 0) in order to avoid incorrectly mixing spin up and down states of the spinor wavefunctions from VASP.

The time-independent tight-binding wavefunctions are

$$H_{mnR} = \langle m0|H|nR\rangle \tag{S2}$$

1.3 Floquet theory

According to the Peierls substitution, under a periodic field $A(r,t)$, the time-dependent tight-binding Hamiltonian should become

$$\begin{aligned}\langle m0|\tilde{H}(t)|nR\rangle &= \langle m0|H|nR\rangle \mathrm{e}^{-\frac{\mathrm{i}e}{\hbar}\int_{\tau_n+R}^{\tau_m} A(r,t)\cdot \mathrm{d}r} \\ &= \langle m0|H|nR\rangle \mathrm{e}^{\frac{\mathrm{i}e}{\hbar}A\cdot(R+\tau_n-\tau_m)}\end{aligned} \tag{S3}$$

where τ_n is the center of the orbital $|n0\rangle$, $e = -|e|$ is the charge of the electron. Here we have assumed that A is spatially uniform.

Then as usual, the Bloch waves can be built from the atomic orbitals with

$$|nk\rangle = \frac{1}{\sqrt{N}}\sum_{R} \mathrm{e}^{\mathrm{i}k\cdot(R+\tau_n)}|nR\rangle \tag{S4}$$

After some algebras, one can obtain the time-dependent Hamiltonian in the basis of Bloch waves as

$$\begin{aligned}\tilde{H}(k,t) &= \langle mk|\tilde{H}(t)|nk\rangle \\ &= \sum_{R} \mathrm{e}^{(k+\frac{e}{\hbar}A)\cdot(R+\tau_n-\tau_m)}\langle m0|H|nR\rangle \\ &= H\left(k+\frac{e}{\hbar}A(t)\right)\end{aligned} \tag{S5}$$

For a A periodic in time with periodicity T, the Fourier transform of $\tilde{H}(k,t)$ should be

$$\begin{aligned}\tilde{H}^m(k) &= \frac{1}{T}\int_0^T \mathrm{e}^{-\mathrm{i}m\omega t}\tilde{H}(k,t)\mathrm{d}t \\ &= \frac{1}{T}\int_0^T \mathrm{e}^{-\mathrm{i}m\omega t} H\left(k + \frac{e}{\hbar}A(t)\right)\mathrm{d}t\end{aligned} \tag{S6}$$

where $\Omega = 2\pi/T$ is the angular frequency. Then an effective Floquet Hamiltonian in the high frequency approximation is [8, 9, 10, 11]

$$H^F_{\text{eff}} = \tilde{H}^0 + \sum_{m\neq 0}\frac{[\tilde{H}^{-m}, \tilde{H}^m]}{2m\hbar\Omega} \tag{S7}$$

One straightforward method for obtaining $\tilde{H}^m(k)$ is to numerically compute the time integral in Eq. (S6). An advantage of this method is that all frequency components can be obtained in the same fashion, and the Floquet Hamiltonian H^F with arbitrary order can be obtained. Another method that is computationally more efficient is to expand Eq. (S6) as

$$\begin{aligned}\tilde{H}(k,t) &= H\left(k + \frac{e}{\hbar}A(t)\right) \\ &= H(k) + \left(\frac{e}{\hbar}\right)\frac{\partial H}{\partial k_i}A_i + \frac{1}{2}\left(\frac{e}{\hbar}\right)^2\frac{\partial^2 H}{\partial k_i \partial k_j}A_i A_j + \cdots\end{aligned} \tag{S8}$$

Assuming that A_i is monochromatic, to obtain a H^F_{eff} up to the order of A^2, we only need to keep the first three terms in Eq. (S8), and $m = 1$ in Eq. (S7). Let

$$\begin{aligned}A_i &= A_i^0\cos(\Omega t + \phi_i) \\ &= A_i^0\frac{\eta_i \mathrm{e}^{\mathrm{i}\Omega t} + \eta_i^* \mathrm{e}^{-\mathrm{i}\Omega t}}{2}\end{aligned} \tag{S9}$$

where $\eta_i = \mathrm{e}^{\mathrm{i}\phi_i}$. One can obtain that

$$\begin{aligned}\tilde{H}^0(k) &= H(k) + \left(\frac{e}{\hbar}\right)^2\sum_{ij}\frac{\partial^2 H}{\partial k_i \partial k_j}\frac{A_i^0 A_j^0}{4}(\eta_i\eta_j^* + \eta_i^*\eta_j) \\ [\tilde{H}^{-1}(k)]^\dagger = \tilde{H}^1(k) &= \left(\frac{e}{\hbar}\right)\sum_i \frac{\partial H}{\partial k_i}\frac{A_i^0}{2}\eta_i\end{aligned} \tag{S10}$$

Note that the Taylor expansion in Eq. (S8) is valid only when $\frac{e}{\hbar}A$ is small as compared with the size of the Brillouin zone, which is on the order of Å^{-1}. With $\omega = 1$ eV and $E = 1$ V/nm, one has $\frac{e}{\hbar}A \approx 0.1\ \text{Å}^{-1}$. Hence the expansion is generally valid.

The interaction between electrons and light is mainly attributed to the $[H^{-1}, H^1]$, which describes the process that a photon is first virtually absorbed, and then virtually emitted by the electron. For a linearly polarized light (LPL), one can easily verify that $[H^{-1}, H^1]$ is zero. Therefore LPL cannot significantly change the electronic structure. Also, LPL preserves the time-reversal symmetry and

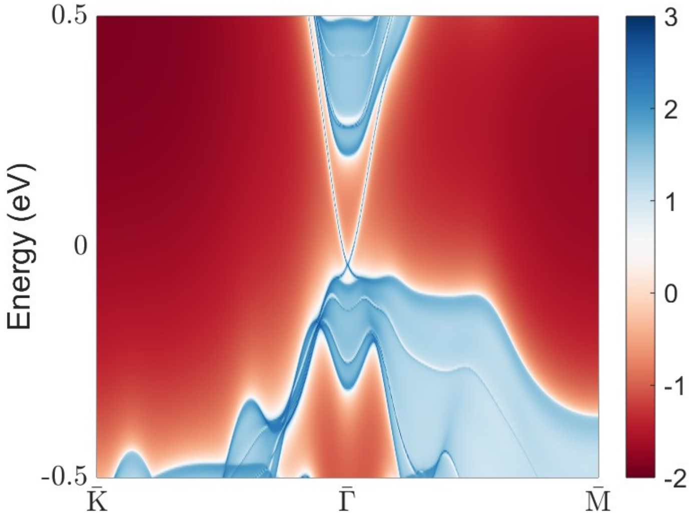

Figure S1: The surface spectrum function of Bi_2Se_3 under linearly polarized light. Note that linearly polarized light cannot open bandgaps on the surface states.

cannot open bandgaps on the surface states of topological materials. This is verified by our calculations (Figure S1).

As discussed in the main text, the model Hamiltonian for the surface states of topological insulators is

$$H_{\mathrm{SS}}(k) = \hbar v_F (k_x \sigma_y - k_y \sigma_x) \tag{S11}$$

Here one has $\frac{\partial H}{\partial k_x} = \hbar v_F \sigma_y$ and $\frac{\partial H}{\partial k_y} = -\hbar v_F \sigma_x$, while all other higher order derivatives of H with respect to k are zero. Thus one has

$$[\tilde{H}^{-1}(k)]^\dagger = \tilde{H}^1(k) = \frac{ev_F}{2}(A_x \eta_x \sigma_y - A_y \eta_y \sigma_x) \tag{S12}$$

For a CPL, one should have $A_x = A_y = A$, $\eta_x = 1$, and $\eta_y = \pm i$. It is not hard to show that

$$[\tilde{H}^{-1}(k), \tilde{H}^1(k)] = \pm e^2 v_F^2 A^2 \sigma_z \tag{S13}$$

Putting Eq. (S13) back to Eq. (S7), one can obtain Eq. (2) in the main text.

2 Responses under light with low frequency

In the main text we used $\Omega = 5$ eV for all the calculations with Bi_2Se_3. Actually, one can (maybe improperly) apply the van Vleck's expansion to light with low frequency. Here we show the results

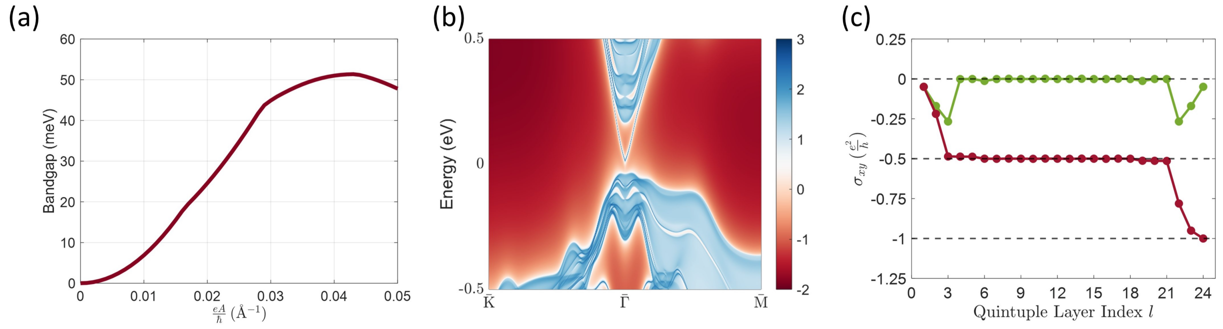

Figure S2: The surface states of Bi_2Se_3 under light with frequency $\Omega = 0.2$ eV. (a) Bandgap v.s. electric field strength at $\Omega = 0.2$ eV. (b) Surface states spectrum at $\Omega = 0.2$ eV and $\frac{eA}{\hbar} = 0.04$ Å^{-1}. (c) Layer-resolved Hall conductance at $\Omega = 0.2$ eV and $\frac{eA}{\hbar} = 0.04$ Å^{-1}.

with $\Omega = 0.2$ eV in Figure S2. One can see that the results are essentially the same as those with $\Omega = 5$ eV; that is, under circularly polarized light a bandgap is opened on the surfaces, and the surfaces acquire a quantized anomalous Hall conductance. Also, at $\Omega = 0.2$ eV, an electric field strength of $\frac{eA}{\hbar} = 0.015$ Å^{-1} ($E = 0.03$ V/nm, $I = 12$ MW/cm^2) would be able to generate a bandgap of 20 meV. On the other hand, at $\Omega = 5$ eV, it takes $E = 5$ V/nm, $I = 3.3 \times 10^6$ MW/cm^2 to generate the same bandgap.

When using the van Vleck's expansion at low frequencies, two sources of errors should be considered: 1) the interband transitions would lead to a non-thermal distribution of the electrons. In other words, one cannot simply use the Fermi-Dirac distribution. As light is turned on, the electrons are gradually pumped from the valence bands to the conduction bands. It takes time for the electron distribution to deviate significantly from the original Fermi-Dirac distribution. Specifically, if the pumping rate is R, then the distribution deviation is $\delta f \propto Rt$. Thus, one should expect that the Fermi-Dirac distribution is valid at the very initial stage ($t \ll 1/R$, which may be a few femtoseconds). 2) there is a correction to the effective Hamiltonian H_{eff} whose relative magnitude is on the order of $\frac{A}{\Omega}$. But this may not make a big difference, because when one uses smaller Ω, then a smaller A would be able to generate the same bandgap, as we described above. Hence, the $\frac{A}{\Omega}$ can be kept relatively small even if Ω is small.

3 Spin Hall Conductivity

We have also calculated the spin Hall conductance based on the Kubo-formula

$$\sigma_{ab}^{s^i} = \frac{e^2}{\hbar} \sum_{n \neq m} \frac{dk}{(2\pi)^2} (f_n - f_m) \frac{\mathrm{Im}\left\{\langle m|j_a^i|n\rangle\langle n|v_b|m\rangle\right\}}{(\omega_m - \omega_n)^2} \tag{S14}$$

Here $j_a^i = \frac{1}{2}(s^i v_a + v_a s^i)$ is the spin current operator. We focus on the spin current with spin-z polarization $\sigma_{ab}^{s^z}$. The layer-resolved spin Hall conductance of a slab system with 24 quintuple layers (QLs) is shown in Figure S3. One can see that the middles layers have spin Hall conductance around $0.36\frac{\hbar}{2e}\frac{e^2}{h}$, which is very close the value when the layer is put in the bulk system (dash horizontal line).

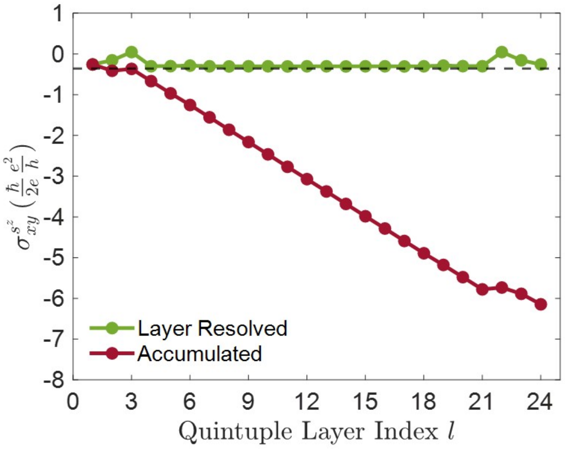

Figure S3: The spin Hall conductance of a Bi_2Si_3 slab system with 24 quintuple layers. The dashed horizontal line is the spin Hall conductance per layer when the layers are put in the bulk system.

In Figure S4 we plot the the spin and charge Hall conductance per layer of bulk Bi_2Si_3. One can see that A field strength of $\frac{eA}{\hbar} = 0.05$ Å^{-1} is not enough to trigger the phase transition to quantum anomalous Hall insulators in the bulk.

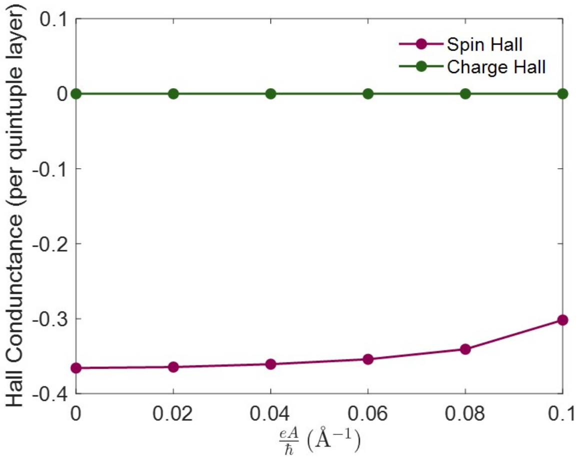

Figure S4: The Hall conductance of a quintuple layer in the bulk Bi_2Si_3 as a function of the strength of the circularly polarized light. A field strength of $\frac{eA}{\hbar} = 0.05$ Å^{-1} is not enough to trigger the phase transition to quantum anomalous Hall insulators in the bulk, thus the charge Hall conductance is always zero. On the other hand, the spin Hall conductance is about $0.36\,\frac{\hbar}{2e}\frac{e^2}{h}$, and slightly varies with the field strength.

References

[1] Hohenberg, P. & Kohn, W. Inhomogeneous electron gas. *Physical review* **136**, B864 (1964).

[2] Kohn, W. & Sham, L. J. Self-consistent equations including exchange and correlation effects. *Physical review* **140**, A1133 (1965).

[3] Kresse, G. & Furthmüller, J. Efficiency of ab-initio total energy calculations for metals and semiconductors using a plane-wave basis set. *Computational materials science* **6**, 15–50 (1996).

[4] Kresse, G. & Furthmüller, J. Efficient iterative schemes for ab initio total-energy calculations using a plane-wave basis set. *Physical review B* **54**, 11169 (1996).

[5] Perdew, J. P., Burke, K. & Ernzerhof, M. Generalized gradient approximation made simple. *Physical review letters* **77**, 3865 (1996).

[6] Blöchl, P. E. Projector augmented-wave method. *Physical review B* **50**, 17953 (1994).

[7] Mostofi, A. A. *et al.* An updated version of wannier90: A tool for obtaining maximally-localised wannier functions. *Computer Physics Communications* **185**, 2309–2310 (2014).

[8] Shirley, J. H. Solution of the schrödinger equation with a hamiltonian periodic in time. *Physical Review* **138**, B979 (1965).

[9] Sambe, H. Steady states and quasienergies of a quantum-mechanical system in an oscillating field. *Physical Review A* **7**, 2203 (1973).

[10] Eckardt, A. Colloquium: Atomic quantum gases in periodically driven optical lattices. *Reviews of Modern Physics* **89**, 011004 (2017).

[11] De Giovannini, U. & Hübener, H. Floquet analysis of excitations in materials. *Journal of Physics: Materials* **3**, 012001 (2019).
